# Racial variation in the advanced prostate cancer genome

**DOI:** 10.1038/s41391-025-00949-w

**Published:** 2025-03-31

**Authors:** Emily M. Feng, Jenny Vo-Phamhi, Aishwarya N. Subramanian, Mikhail Dias, Adam Foye, Jake Vinson, Julian C. Hong, Stephen J. Freedland, Joshi J. Alumkal, Himisha Beltran, Colm Morrissey, Peter S. Nelson, Arul M. Chinnaiyan, Rahul Aggarwal, Eric J. Small, David A. Quigley, Martin Sjöström, Shuang G. Zhao, William S. Chen

**Affiliations:** 1 Helen Diller Family Comprehensive Cancer Center, University of California San Francisco, San Francisco, CA USA; 2 Department of Radiation Oncology, University of California San Francisco, San Francisco, CA USA; 3 Columbia University Vagelos College of Physicians and Surgeons, New York, NY USA; 4 Department of Medicine, University of California San Francisco, San Francisco, CA USA; 5 Prostate Cancer Clinical Trials Consortium (PCCTC), New York, NY USA; 6 Department of Urology, Cedars-Sinai Medical Center, Los Angeles, CA USA; 7 Section of Urology, Durham VA Medical Center, Durham, NC USA; 8 Department of Internal Medicine, Rogel Cancer Center, University of Michigan, Ann Arbor, MI USA; 9 Department of Medical Oncology, Dana-Farber Cancer Institute and Harvard Medical School, Boston, MA USA; 10 Department of Urology, University of Washington School of Medicine, Seattle, WA USA; 11 Division of Human Biology, Fred Hutchinson Cancer Center, Seattle, WA USA; 12 Division of Clinical Research, Fred Hutchinson Cancer Center, Seattle, WA USA; 13 Department of Medicine, University of Washington School of Medicine, Seattle, WA USA; 14 Departments of Pathology and Urology, University of Michigan, Ann Arbor, MI USA; 15 Department of Urology, University of California San Francisco, San Francisco, CA USA; 16 Department of Epidemiology and Biostatistics, University of California San Francisco, San Francisco, CA USA; 17 Department of Clinical Sciences Lund, Division of Oncology, Lund University, Lund, Sweden; 18 Department of Hematology, Oncology and Radiation Physics, Skåne University Hospital, Lund, Sweden; 19 Department of Human Oncology, University of Wisconsin-Madison, Madison, WI USA; 20 Carbone Cancer Center, University of Wisconsin-Madison, Madison, WI USA

**Keywords:** Cancer genetics, Prostate cancer

## Abstract

**Background:**

Racial differences in metastatic castration-resistant prostate cancer (mCRPC) genomes have not yet been fully studied. We aimed to investigate transcriptomic, mutational, and clinical differences by race in a large multi-institutional cohort of men with mCRPC.

**Methods:**

Genomic and clinicopathologic data from four mCRPC tumor biopsy cohorts were obtained and aggregated. Gene set enrichment analyses were performed to assess pathway-level differences in gene expression by patient race. DNA alteration frequencies of known prostate cancer driver genes and clinical outcomes were compared across racial groups.

**Results:**

In our cohort of 445 men with mCRPC, tumors from African American patients (*N* = 26) demonstrated higher expression of *MYC* pathway genes (FDR *q* = 0.03) and lower expression of IFN-γ, IL-6/JAK/STAT3, and inflammatory pathway genes (FDR *q* < 0.001) compared to tumors from European American patients. *TMPRSS2:ERG* gene fusions were observed more frequently in tumors from European American compared to African American patients (41% vs. 11%, *P* = 0.015). Asian patients (*N* = 9) and other racial groups comprised a small minority of our cohort. No differences in overall survival were noted across racial groups.

**Conclusions:**

Despite demonstrating similar clinical outcomes, cancers from African Americans display distinct tumor biology. Specifically, we observed racial differences in expression of prostate cancer driver gene pathways (including potential clinically actionable pathways of IFN-γ and JAK/STAT) and DNA alterations, including *TMPRSS2:ERG* gene fusion. Our findings highlight the importance of racial diversity in future genomic profiling and clinical trials efforts.

## Introduction

Racial differences in the clinical and genomic characteristics of prostate cancer (PCa) have been previously reported [1–5]. At the population level, African American men have been shown to have higher rates of PCa incidence and cancer-specific mortality than European American men, whereas Asian men have been shown to have generally lower-risk disease [2, 6–8]. Differences in tumor genomics by race have also been observed, such as higher rates of *TMPRSS2:ERG* gene fusions in European American men compared to non-European American men [3] and differing mutation rates of driver genes such as *TP53*, *AR*, and *CDK12* [1, 4]. However, most of these genomic findings are from patients with localized disease. The relationships between race, tumor genomics, and clinical outcomes have not yet been fully explored in metastatic castration-resistant prostate cancer (mCRPC), the most advanced and lethal form of PCa. Several large genomic studies on race have interrogated mutations through targeted panels [1, 4, 5], but few integrated copy number and structural variant data to provide a more comprehensive view of gene alteration status. Moreover, DNA alterations have generally not been examined alongside paired gene expression data to provide complementary insights into tumor biology. In this study, we investigated transcriptomic, mutational, and clinical differences by race in patients with mCRPC.

## Materials/subjects and methods

We aggregated gene expression data from four mCRPC cohorts, as previously described [9, 10]. The four cohorts were from the Fred Hutchinson Cancer Center (FHCC; *N* = 63) [11], Weill Cornell Medicine (WCM; *N* = 27) [12], Stand Up 2 Cancer / Prostate Cancer Foundation East Coast Dream Team (ECDT; *N* = 255) [13], and West Coast Dream Team (WCDT; *N* = 136) [14, 15]. For patients with multiple mCRPC biopsy timepoints, the first timepoint was included. Normalized gene expression, mutation calls, and copy number calls for the FHCC, WCM, and ECDT cohorts were obtained directly from cBioPortal [16]. WCDT genomic and clinical data were obtained from a prior publication [10]. For the FHCC cohort, RNA expression profiling was performed using the Agilent 44 K whole human genome expression kit, copy number profiling was performed using microarray comparative genomic hybridization (CGH) using GISTIC 2.0, and whole exome sequencing was performed using the Illumina Hiseq 2000 with either 50 bp or 100 bp paired end sequences on either the Nimblegen V2 or V3 platforms [17]. For the WCM cohort, DNA profiling was performed using the Illumina HiSeq platform with an intended mean-target exome coverage of 100x, and MuTect [18], Oncotator [19], and MutSig [20] were applied for variant calling. For the ECDT cohort, RNA profiling was performed using polyA+ RNA isolation or Agilent SureSelect Human All Exon V4 [13, 21], DNA profiling was performed using the AllPrep DNA/RNA/miRNA kit (QIAGEN), and variant calling was performed using the MuTect and log-2 exon coverage-based pipelines [18, 22]. For the WCDT cohort, RNA-seq was performed on an Illumina NextSeq500 in 2 × 76 bp paired-end runs using the Agilent Absolutely RNA Nano Prep kit, DNA (whole-genome) sequencing was performed on an Illumina profiler, and variant calling was performed using the Strelka [23], MuTect [18], Manta [24], and CopyCat [25] methods.

RNA expression batch correction was performed by first converting gene expression levels into a per-sample gene rank to standardize genes across cohorts and then applying an empirical bayes framework for batch correction, as previously described [10]. Clinical and pathologic variables were obtained from the original publications of these cohorts. Pathway scores using the Hallmark Pathways from MSigDb [26] were calculated using the published gene set enrichment analysis (GSEA) tool, with a prespecified significance level of FDR *q* < 0.05 [27]. Wilcoxon rank-sum testing was performed to assess for group differences by race of gene signature scores for select pathways (*AR* activity, neuroendocrine differentiation) known to be biological drivers of prostate cancer. Oncogene-activating alterations (amplification and/or mutation) and tumor-suppressor bi-allelic inactivating alterations (copy-number loss and/or mutation) were defined as previously described [14, 15]. Fisher’s exact test was used to evaluate groupwise differences in the proportions of categorical variables. PSA50 response to androgen receptor pathway inhibitor (ARPI) treatment was defined as a 50% or greater reduction in PSA compared to baseline. Overall survival analysis was performed using the Kaplan-Meier method with Cox proportional hazards testing for significance. All independence and hypothesis tests were performed using a two-sided significance level of 0.05.

## Results

Tumor biopsies from 481 men with mCRPC were profiled using whole-transcriptome RNAseq. Of these 481 patients, 445 had self-reported race or ethnic data available and were selected for further analysis. 399 of 445 patients (90%) self-identified as white or European American / Caucasian, 26 (6%) identified as Black or African American, and 9 (2.0%) identified as Asian. The six patients who self-identified as multiracial, 3 as “white or non-white Hispanic,” and 2 as Native American were excluded from statistical analysis due to low sample sizes. 388 patients with known race had paired DNA-sequencing data available. Across the Asian, African American, and European American racial groups, no significant differences were observed with respect to age, tumor histology, or biopsy site (*P* > 0.05; Table 1).

**Table 1:** Patient clinicopathologic features.

Characteristic	European American (*N* = 399)	Black / AA (*N* = 26)	Asian (*N* = 9)	*P*-value
Histology				0.47
Adenocarcinoma	302 (76)	18 (69)	8 (89)	
Small cell / neuroendocrine	30 (8)	3 (12)	1 (11)	
Missing	67 (17)	5 (19)	0 (0)	
Prior exposure to ASI therapy				0.24
ASI naive	148 (37)	11 (42)	6 (67)	
ASI exposed	175 (44)	11 (42)	1 (11)	
Missing	76 (19)	4 (15)	2 (22)	
Metastatic biopsy site				0.7
Bone	129 (32)	7 (27)	1 (11)	
Lymph node	153 (38)	11 (42)	5 (55)	
Liver	61 (15)	5 (19)	3 (33)	
Other	38 (10)	3 (12)	0 (0)	
Missing	3 (1)	0 (0)	0 (0)	

All clinicopathologic variables were measured at time of biopsy and are presented as “Number (%).” *P*-values represent comparison between European American, Black / African American (AA), and Asian groups.

**Fig. 1:**
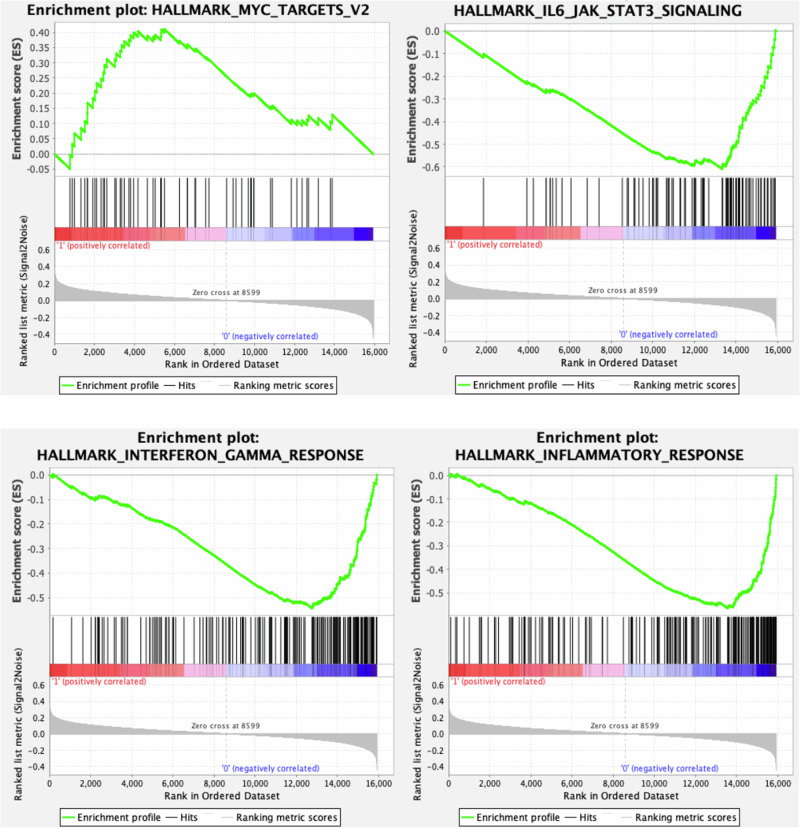
GSEA plots demonstrating overexpression of MYC pathway genes (top left) and under-expression of IL6/JAK/STAT3 (top right), IFN-γ (bottom left), and inflammatory pathway genes (bottom right) in African American compared to European American patients. Depicted within each plot, from top to bottom: gene set enrichment score (top), gene ranked order (middle), and gene ranking metric score (bottom).

We first examined racial differences in tumor gene expression by performing pathway-level gene set enrichment analysis (GSEA) to compare racial groups pairwise, specifying European American as the reference group. Examining all MSigDb Hallmark pathways, GSEA identified African American patients as having higher expression of *MYC* pathway genes (NES = 1.54, *P* = 0.013, FDR *q* = 0.036) and lower expression of IFN-γ (NES = −2.52, *P* < 0.001, FDR *q* < 0.001), IL-6/JAK/STAT3 (NES = −2.50, *P* < 0.001, FDR *q* < 0.001), and inflammatory pathway genes (NES = −2.64, *P* < 0.001, FDR *q* < 0.001) compared to European American patients (Fig. 1). GSEA comparing Asian to European American patients revealed higher expression of epithelial-to-mesenchymal transition (NES = 2.73), TGFβ-signaling (NES = 1.69), hypoxia (NES = 2.42), and NF-kB/TNF-α signaling genes (NES = 1.71) in tumors of Asian compared to European American patients (*P* < 0.001, FDR *q* < 0.01 for all; Fig. S1). Altogether, these findings highlighted a diversity of oncogenic driver pathways in mCRPC and suggested transcriptomic differences by race. GSEA revealed several additional pathways to be differentially expressed by race (Table S1). However, expression of specific transcriptomic signatures well-known in PCa, including previously published gene signatures for *AR* activity and neuroendocrine differentiation [12, 28, 29], were assessed and were found not to be significantly different across racial groups (*P* > 0.05 for all).

**Fig. 2:**
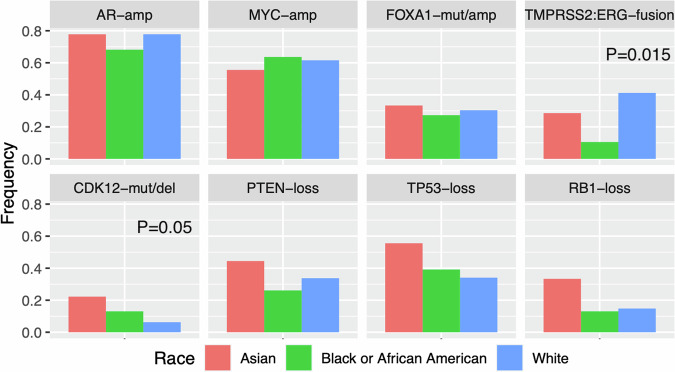
Barplots showing observed frequencies of DNA alterations in select prostate cancer driver genes, stratified by race. “amp” = amplification; “mut” = mutation; “del” = deletion, “loss” = 2 DNA alterations or deep deletion. *P* > 0.05 across racial groups unless otherwise specified.

We then assessed for differences in the DNA alteration frequencies of PCa driver genes across racial groups (Fig. 2). *AR* and *MYC* amplification were observed in the majority of tumors and at similarly high rates across groups (*P* > 0.05). *TMPRSS2:ERG* gene fusions were observed more commonly in tumors from European American compared to African American patients (41% vs. 11%, *P* = 0.015). Rates of *CDK12* alterations in Asian, African American, and European American patients were 22%, 13%, and 6% respectively in our cohort (*P* = 0.05), consistent with prior reports [4]. Prevalence of *FOXA1* alterations and biallelic loss of *PTEN*, *RB1*, and *TP53* were nominally highest in tumors from Asian patients in our cohort, though these differences were not statistically significant (*P* > 0.05).

197 of 481 patients had mature overall survival outcomes available and 80 had data available on PSA50 response to ARPI treatment. PSA50 response to ARPI treatment was higher in ARPI-naïve compared to ARPI-exposed patients (*P* < 0.001). No racial differences were observed with respect to PSA50 response to ARPI treatment when stratifying analysis by prior ARPI exposure (*P* > 0.05). Overall survival was similarly poor across racial groups, with an observed cohort-wide median survival of 20.9 months (*P* = 0.9; Fig. S2).

## Discussion

We used whole-transcriptome RNA-seq of metastasis biopsies to identify oncogenic pathways differentially expressed across racial groups and analyzed DNA-seq performed on paired biopsies to assess differences in driver gene alterations by race.

Notably, despite similar clinical outcomes and response to treatments, we observed important biological differences suggesting tumors from African American and European American patients are distinct. Consistent with prior reports, we identified a significantly lower frequency of *TMPRSS2:ERG* fusions in tumors from African American patients compared to European American patients. *MYC* amplification was also most frequently observed in African American patients. *FOXA1* and *CDK12* alteration frequencies stratified by race similarly mirror prior studies, which reported higher rates in Asians compared to other racial groups [4, 30]. Asian patients in our study also demonstrated the highest frequency of *PTEN*, *TP53*, and *RB1* tumor suppressor loss, though this enrichment was not statistically significant. However, given the limited number of Asian patients in the present study, future larger studies are needed including tumor genomic studies of Asian patients in Asia.

Our comparison of tumor transcriptomes revealed differences by race, bearing potential translational implications. For example, tumors from African American patients demonstrated lower expression of IFN-γ and IL-6/JAK/STAT3 pathway genes than tumors from European American patients. IFN-γ treatment was recently shown to increase sensitivity to taxane-based chemotherapy in preclinical mCRPC models [31]. Additionally, JAK-inhibitor therapy has emerged as a promising therapeutic strategy to combat ARPI resistance and halt mCRPC progression [32, 33], and a phase II study is now ongoing to evaluate the effect of pacritinib (JAK2 inhibitor) in biochemically recurrent prostate cancer [34]. Race-based differences in IFN-γ and JAK/STAT-pathway activity highlight the need to enroll diverse study populations in upcoming trials to adequately evaluate the safety and efficacy of these novel therapies. Whether pathway activity is predictive of response to targeted therapies remains to be seen.

A limitation of this study was the unavailability of detailed information on other social determinants of health (SDOH). We could not interrogate additional factors potentially associated with race in our cohort (e.g. structural racism, income, lifestyle factors). Future studies are needed to further elucidate SDOH potentially driving racial differences in tumor biology. Another limitation was the low number of non-European American patients. With respect to sample size, we aggregated cohorts from four different institutions to perform the largest study of its kind in mCRPC. Genomic data are especially challenging to collect for a cohort of this size, considering prostate cancer metastases are not routinely biopsied in clinical practice. Nevertheless, only 10% of our patients were non-European American, including a total of 56 African American and nine Asian patients. Additional studies are thus needed to validate such findings as the lack of difference in clinical outcomes across racial groups. The number of patients who identified as Native American (*N* = 2) was too low to conduct even exploratory analyses, highlighting the need for improved outreach. Racial diversity is a well-known challenge in clinical research, with issues ranging from inconsistent data reporting to selection biases [35, 36]. Collaborative efforts to include more underrepresented minorities in future studies are needed to develop better personalized cancer therapies.

## Conclusions

Despite similar clinical outcomes, we identified key transcriptomic and genomic differences in mCRPC tumors by race. Specifically, by integrating tumor DNA profiling with gene expression profiling, we identified racial differences in the expression and DNA-alteration frequencies of mCRPC driver genes including IFN-γ and JAK/STAT. Our findings highlight the importance of racial diversity in future genomic profiling and translational research efforts.

## Supplementary information

Supplementary Table

Supplemental Figures

## Data Availability

DNA alteration, DNA methylation, and RNA-seq data are available at dbGAP (phs001648). Clinical data are publicly available as described in the Methods section of this study. Additional data relevant to this manuscript are available upon request.

## References

[1] Mahal BA, Alshalalfa M, Kensler KH, Chowdhury-Paulino I, Kantoff P, Mucci LA, et al. Racial differences in genomic profiling of prostate cancer. N. Engl J Med. 2020;383:1083–5. 32905685 10.1056/NEJMc2000069 PMC8971922

[2] Mahal BA, Gerke T, Awasthi S, Soule HR, Simons JW, Miyahira A, et al. Prostate cancer racial disparities: a systematic review by the prostate cancer foundation panel. Eur Urol Oncol. 2022;5:18–29. 34446369 10.1016/j.euo.2021.07.006

[3] Zhou CK, Young D, Yeboah ED, Coburn SB, Tettey Y, Biritwum RB, et al. TMPRSS2:ERG Gene Fusions in Prostate Cancer of West African Men and a meta-analysis of racial differences. Am J Epidemiol. 2017;186:1352–61. 28633309 10.1093/aje/kwx235 PMC5860576

[4] Sivakumar S, Lee JK, Moore JA, Hopkins J, Newberg JY, Madison R, et al. Comprehensive genomic profiling and treatment patterns across ancestries in advanced prostate cancer: a large-scale retrospective analysis. Lancet Digital Health. 2023;5:e380–9. 37236698 10.1016/S2589-7500(23)00053-5

[5] Stopsack KH, Nandakumar S, Arora K, Nguyen B, Vasselman SE, Nweji B, et al. Differences in prostate cancer genomes by self-reported race: contributions of genetic ancestry, modifiable cancer risk factors, and clinical factors. Clin Cancer Res. 2022;28:318–26. 34667026 10.1158/1078-0432.CCR-21-2577 PMC8776579

[6] Vidal AC, Oyekunle T, Feng T, Freedland AR, Moreira D, Castro-Santamaria R, et al. Asian race and risk of prostate cancer: results from the REDUCE Study. Cancer Epidemiol, Biomark Prev. 2020;29:2165–70. 10.1158/1055-9965.EPI-20-0646 32856605

[7] Gong J, Kim DM, Freeman MR, Kim H, Ellis L, Smith B, et al. Genetic and biological drivers of prostate cancer disparities in Black men. Nat Rev Urol. 2024;21:274–89. 37964070 10.1038/s41585-023-00828-w

[8] Gong J, Kim DM, De Hoedt AM, Bhowmick N, Figlin R, Kim HL, et al. Disparities with systemic therapies for black men having advanced prostate cancer: where do we stand? J Clin Oncol. 2024;42:228–36. 37890125 10.1200/JCO.23.00949 PMC10824384

[9] Feng E, Rydzewski NR, Zhang M, Lundberg A, Bootsma M, Helzer KT, et al. Intrinsic molecular subtypes of metastatic castration-resistant prostate cancer. Clin Cancer Res. 2022;28:5396–404. 36260524 10.1158/1078-0432.CCR-22-2567 PMC9890931

[10] Aggarwal R, Rydzewski NR, Zhang L, Foye A, Kim W, Helzer KT, et al. Prognosis associated with luminal and basal subtypes of metastatic prostate cancer. JAMA Oncol. 2021;7:1644. 34554200 10.1001/jamaoncol.2021.3987 PMC8461554

[11] Kumar A, Coleman I, Morrissey C, Zhang X, True LD, Gulati R, et al. Substantial interindividual and limited intraindividual genomic diversity among tumors from men with metastatic prostate cancer. Nat Med. 2016;22:369–78. 26928463 10.1038/nm.4053 PMC5045679

[12] Beltran H, Prandi D, Mosquera JM, Benelli M, Puca L, Cyrta J, et al. Divergent clonal evolution of castration-resistant neuroendocrine prostate cancer. Nat Med. 2016;22:298–305. 26855148 10.1038/nm.4045 PMC4777652

[13] Abida W, Cyrta J, Heller G, Prandi D, Armenia J, Coleman I, et al. Genomic correlates of clinical outcome in advanced prostate cancer. Proc Natl Acad Sci USA. 2019;116:11428–36. 31061129 10.1073/pnas.1902651116 PMC6561293

[14] Chen WS, Aggarwal R, Zhang L, Zhao SG, Thomas GV, Beer TM, et al. Genomic drivers of poor prognosis and enzalutamide resistance in metastatic castration-resistant prostate cancer. Eur Urol. 2019;76:562–71. 30928160 10.1016/j.eururo.2019.03.020 PMC6764911

[15] Quigley DA, Dang HX, Zhao SG, Lloyd P, Aggarwal R, Alumkal JJ, et al. Genomic hallmarks and structural variation in metastatic prostate cancer. Cell. 2018;174:758–69.e9. 30033370 10.1016/j.cell.2018.06.039 PMC6425931

[16] Cerami E, Gao J, Dogrusoz U, Gross BE, Sumer SO, Aksoy BA, et al. The cBio cancer genomics portal: an open platform for exploring multidimensional cancer genomics data: Fig. 1. Cancer Discov. 2012;2:401–4. 22588877 10.1158/2159-8290.CD-12-0095 PMC3956037

[17] Mermel CH, Schumacher SE, Hill B, Meyerson ML, Beroukhim R, Getz G. GISTIC2.0 facilitates sensitive and confident localization of the targets of focal somatic copy-number alteration in human cancers. Genome Biol. 2011;12:R41. 21527027 10.1186/gb-2011-12-4-r41 PMC3218867

[18] Cibulskis K, Lawrence MS, Carter SL, Sivachenko A, Jaffe D, Sougnez C, et al. Sensitive detection of somatic point mutations in impure and heterogeneous cancer samples. Nat Biotechnol. 2013;31:213–9. 23396013 10.1038/nbt.2514 PMC3833702

[19] Ramos AH, Lichtenstein L, Gupta M, Lawrence MS, Pugh TJ, Saksena G, et al. Oncotator: cancer variant annotation tool. Hum Mutat. 2015;36:E2423–E2429. 25703262 10.1002/humu.22771 PMC7350419

[20] Lawrence MS, Stojanov P, Polak P, Kryukov GV, Cibulskis K, Sivachenko A, et al. Mutational heterogeneity in cancer and the search for new cancer-associated genes. Nature. 2013;499:214–8. 23770567 10.1038/nature12213 PMC3919509

[21] Robinson D, Van Allen EM, Wu Y-M, Schultz N, Lonigro RJ, Mosquera J-M, et al. Integrative clinical genomics of advanced prostate cancer. Cell. 2015;161:1215–28. 26000489 10.1016/j.cell.2015.05.001 PMC4484602

[22] Lonigro RJ, Grasso CS, Robinson DR, Jing X, Wu Y-M, Cao X, et al. Detection of somatic copy number alterations in cancer using targeted exome capture sequencing. Neoplasia. 2011;13:1019–IN21. 22131877 10.1593/neo.111252 PMC3223606

[23] Saunders CT, Wong WSW, Swamy S, Becq J, Murray LJ, Cheetham RK. Strelka: accurate somatic small-variant calling from sequenced tumor–normal sample pairs. Bioinformatics. 2012;28:1811–7. 22581179 10.1093/bioinformatics/bts271

[24] Chen X, Schulz-Trieglaff O, Shaw R, Barnes B, Schlesinger F, Källberg M, et al. Manta: rapid detection of structural variants and indels for germline and cancer sequencing applications. Bioinformatics. 2016;32:1220–2. 26647377 10.1093/bioinformatics/btv710

[25] Miller CA, Gindin Y, Lu C, Griffith OL, Griffith M, Shen D, et al. Aromatase inhibition remodels the clonal architecture of estrogen-receptor-positive breast cancers. Nat Commun. 2016;7:12498. 27502118 10.1038/ncomms12498 PMC4980485

[26] Liberzon A, Birger C, Thorvaldsdóttir H, Ghandi M, Mesirov JP, Tamayo P. The molecular signatures database hallmark gene set collection. Cell Syst. 2015;1:417–25. 26771021 10.1016/j.cels.2015.12.004 PMC4707969

[27] Subramanian A, Tamayo P, Mootha VK, Mukherjee S, Ebert BL, Gillette MA, et al. Gene set enrichment analysis: a knowledge-based approach for interpreting genome-wide expression profiles. Proc Natl Acad Sci. 2005;102:15545–50. 16199517 10.1073/pnas.0506580102 PMC1239896

[28] Bluemn EG, Coleman IM, Lucas JM, Coleman RT, Hernandez-Lopez S, Tharakan R, et al. Androgen receptor pathway-independent prostate cancer is sustained through FGF signaling. Cancer Cell. 2017;32:474–89.e6. 29017058 10.1016/j.ccell.2017.09.003 PMC5750052

[29] Aggarwal R, Huang J, Alumkal JJ, Zhang L, Feng FY, Thomas GV, et al. Clinical and genomic characterization of treatment-emergent small-cell neuroendocrine prostate cancer: a multi-institutional prospective study. J Clin Oncol. 2018;36:2492–503. 29985747 10.1200/JCO.2017.77.6880 PMC6366813

[30] Pan J, Zhao J, Ni X, Gan H, Wei Y, Wu J, et al. The prevalence and prognosis of next‐generation therapeutic targets in metastatic castration‐resistant prostate cancer. Mol Oncol. 2022;16:4011–22. 36209367 10.1002/1878-0261.13320 PMC9718110

[31] Korentzelos D, Wells A, Clark AM. Interferon-γ increases sensitivity to chemotherapy and provides immunotherapy targets in models of metastatic castration-resistant prostate cancer. Sci Rep. 2022;12:6657. 35459800 10.1038/s41598-022-10724-9 PMC9033763

[32] Chan JM, Zaidi S, Love JR, Zhao JL, Setty M, Wadosky KM, et al. Lineage plasticity in prostate cancer depends on JAK/STAT inflammatory signaling. *Science* eabn0478 (2022) 10.1126/science.abn0478. 10.1126/science.abn0478 PMC9653178 35981096

[33] Udhane V, Maranto C, Hoang DT, Gu L, Erickson A, Devi S, et al. Enzalutamide-induced feed-forward signaling loop promotes therapy-resistant prostate cancer growth providing an exploitable molecular target for Jak2 inhibitors. Mol Cancer Ther. 2020;19:231–46. 31548294 10.1158/1535-7163.MCT-19-0508 PMC6946850

[34] Kilari D, Szabo A, Hall WA, Nelson AA, Johnson S, Giever TA, et al. A single-arm, open-label, phase 2 study evaluating pacritinib for patients with biochemical recurrence after definitive treatment for prostate cancer: Blast study. JCO. 2022;40:TPS220–TPS220.

[35] Alegria M, Sud S, Steinberg BE, Gai N, Siddiqui A. Reporting of Participant Race, Sex, and Socioeconomic Status in Randomized Clinical Trials in General Medical Journals, 2015 vs 2019. JAMA Netw Open. 2021;4:e2111516. 34037736 10.1001/jamanetworkopen.2021.11516 PMC8155820

[36] Brady L, Lee JR, Yu EY, Lin D, Gore JL, Nelson PS, et al. Determining clinical perspectives and strategies for improving enrollment of minoritized communities in prostate cancer clinical trials. Am J Clin Exp Urol. 2023;11:385–94. 37941652 PMC10628627

