# Supplementary material for: Racial variation in the advanced prostate cancer genome
Source: Prostate Cancer Prostatic Dis. 2025 Mar 31;28(4):902–7. doi: 10.1038/s41391-025-00949-w (PMC12643939; doi:10.1038/s41391-025-00949-w)
Supplement: Supplementary file 2 — Supplemental Figures [file 41391_2025_949_MOESM2_ESM.pdf]

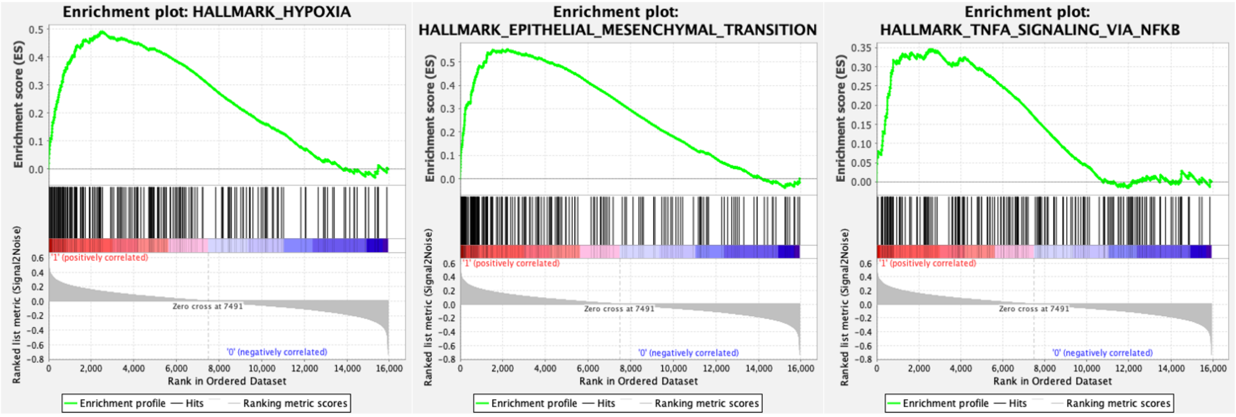

Figure S1. GSEA plots demonstrating overexpression of hypoxia (left), EMT (middle), and NF-kB/TNF-pathway genes (right) in Asian compared to European American patients.

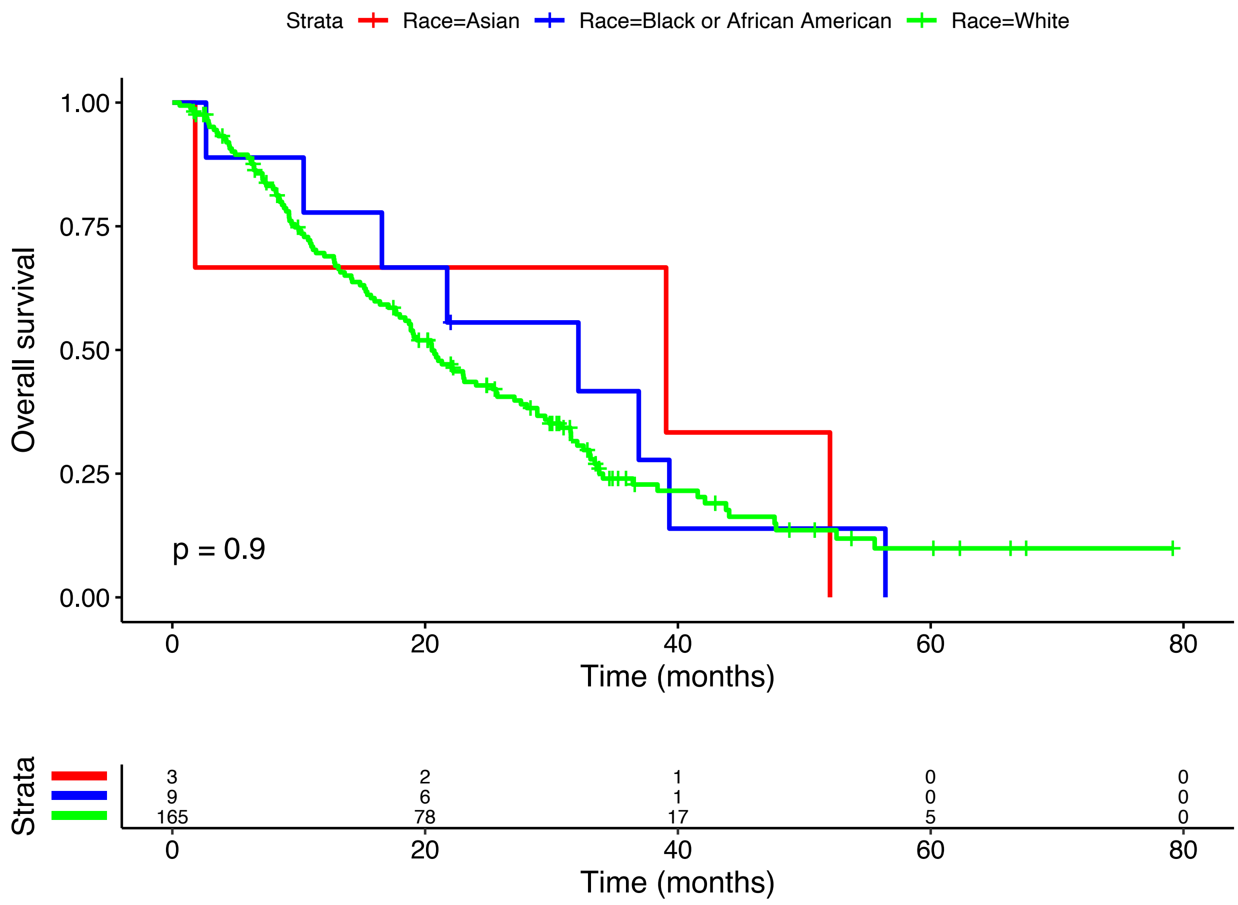

Figure S2. Kaplan-Meier plot showing overall survival, stratified by patient race.
